# What Do We Know About Medical Cannabis in Neurological Disorders and What Are the Next Steps?

**DOI:** 10.3389/fphar.2022.883987

**Published:** 2022-04-27

**Authors:** Clémence Lacroix, Isabelle Alleman-Brimault, Arnaud Zalta, Frank Rouby, Catherine Cassé-Perrot, Elisabeth Jouve, Laurence Attolini, Romain Guilhaumou, Joëlle Micallef, Olivier Blin

**Affiliations:** APHM, INSERM, Inst Neurosci Syst, UMR 1106, Aix Marseille Univ, University Hospital Federation DHUNE, Service de Pharmacologie Clinique et Pharmacovigilance, Marseille, France

**Keywords:** medical cannabis, neurological disorders, amyotrophic lateral sclerosis, Parkinson, Alzheimer, pharmacology, scientific research

## Abstract

Medical use of cannabis has been receiving growing attention over the last few decades in modern medicine. As we know that the endocannabinoid system is largely involved in neurological disorders, we focused on the scientific rationale of medical cannabis in three neurological disorders: amyotrophic lateral sclerosis, Parkinson’s disease, and Alzheimer’s disease through pharmacological plausibility, clinical studies, and patients’ view. Clinical studies (randomized controlled trials, open-label studies, cohorts, and case reports) exploring medical cannabis in these disorders show different results depending on the methods and outcomes. Some show benefits on motor symptoms and others on non-motor symptoms and quality of life. Concerning patients’ view, several web surveys were collected, highlighting the real use of cannabis to relieve symptoms of neurological disorders, mostly outside a medical pathway. This anarchic use keeps questioning particularly in terms of risks: consumption of street cannabis, drug–drug interactions with usual medical treatment, consideration of medical history, and adverse reactions (psychiatric, respiratory, cardiovascular disorders, etc.), underlining the importance of a medical supervision. To date, most scientific data support the therapeutic potential of cannabis in neurological disorders. As far as patients and patients’ associations are calling for it, there is an urgent need to manage clinical studies to provide stronger evidence and secure medical cannabis use.

## Introduction

Medical use of cannabis has been receiving growing attention over the last few decades in modern medicine. As cannabis is a complex plant containing hundreds of cannabinoids, we keep questioning about its therapeutic benefits, justified by its pleiotropic pharmacological activity. As a result, it has been reported that changes in endocannabinoid levels may be related to neurological diseases such as Parkinson’s disease, Huntington’s disease, Alzheimer’s disease, and multiple sclerosis (Fraguas-Sánchez and Torres-Suárez, 2018). As we know that the endocannabinoid system (ECS) is largely involved in neurological disorders, we here chose to focus on the scientific rationale of medical cannabis through a narrative review in three neurological disorders: amyotrophic lateral sclerosis (ALS), Parkinson’s disease (PD), and Alzheimer’s disease (AD) through pharmacological plausibility, clinical studies, and patients’ view. Developing medical cannabis could be an important issue to better control neurodegeneration.

## Pharmacological Plausibility

ECS is largely expressed in the cerebellum, basal ganglia, and hippocampus and is thus an area of choice for molecular targets. Characterization of the ECS and detection of widespread cannabinoid receptors in the brain and peripheral tissues have opened the door to a vast field of research. The ECS is formed by cannabinoid receptors 1 and 2 (CB1 and CB2), the two endocannabinoids anandamide and 2-arachidonoylglycerol, and endocannabinoid anabolic and catabolic enzymes. Manipulation of the ECS may have beneficial disease-modifying potential in neurological disorders. Exogenous cannabinoids play a pleiotropic activity mostly through two cannabinoid receptors: CB1 is predominantly expressed in the brain, and CB2 is primarily found in the cells of the immune system (Lucas et al., 2018). Since the pathophysiology of motor neuron degeneration in ALS may involve mitochondrial dysfunction, excessive glutamate activity, oxidative stress, neuroinflammation, and growth factor deficiency, cannabis could be effective in modulating these processes (Bilsland and Greensmith, 2008; Carter et al., 2010; Papadimitriou et al., 2010; Appel et al., 2011). To support these hypotheses, a recent meta-analysis of preclinical studies in murine ALS models conducted by Urbi and colleagues suggests that cannabinoid receptor agonists may improve survival time (Urbi et al., 2019b).

PD mostly involves dopaminergic and cholinergic systems. The interactions between cannabinoids and dopamine in the basal ganglia may involve both the modulation of other neurotransmitters (GABA, glutamate) and the activation of CB1 and CB2 (Stampanoni Bassi et al., 2017; Patricio et al., 2020). Preclinical studies in the animal model of PD have shown various influences of cannabis on motor and non-motor behaviors: reducing motor fluctuations and levodopa-induced dyskinesias (Segovia et al., 2003; Morgese et al., 2007; Song et al., 2014). Activation of CB2 has shown a reduction in dopamine depletion in PD rats (García-Arencibia et al., 2007). In a preclinical study investigating the role of a CB2 receptor agonist on 1-methyl-4-phenyl-1,2,3,6-tetrahydropyridine (MPTP)-induced neurotoxicity in a mouse model of PD managed in 2017, the use of a CB2 agonist reversed the depletion of CB2 and thus increased the levels of dopamine and improved the behavior of PD mice (Shi et al., 2017). Cannabinoids seem to be protective by binding to the CB1 receptor, inhibiting the dopamine beta hydroxylase activity and decreasing glutamate levels or by binding to CB2, reducing neuroinflammation (Ferreira et al., 2020). All these considerations suggest therapeutic benefits of cannabis in PD.

AD is characterized by extracellular deposits of β-amyloid plaques and neurofibrillary tangles of tau protein (Selkoe, 2011). Cannabis promotes neuroprotection through different signal pathways mediated indirectly by CB receptors by reducing the β-amyloid peptide action and tau phosphorylation, as well as modulating oxidative stress and inflammation (Esposito et al., 2006; Aso and Ferrer, 2014). CB1 and CB2 agonists ameliorated memory and cognitive impairment in mice that have received intracerebral injection of β-amyloid peptide (Ramirez, 2005). CB2 activation also reduced levels of neurotoxic factors and pro-inflammatory mediators produced by reactive astrocytes and microglial cells, stimulated microglial proliferation and migration, and decreased β-amyloid peptide levels (Cristino et al., 2020). To resume, cannabis improved immune function, amyloidogenesis, and reduced behavioral symptoms and pain but also stimulated appetite and inhibited acetylcholinesterase in animal models of AD (Cooray et al., 2020; Li et al., 2020).

These pharmacological considerations concerning cannabis in neurological disorders suggest mechanism-based therapeutic targets for future clinical studies.

## Clinical Studies

**FIGURE 1:**
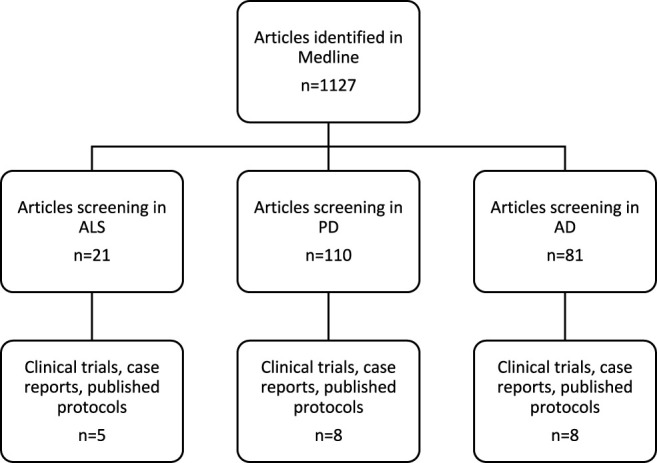
Flow chart of the included clinical studies.

We managed a literature search on Medline using the keywords “medical cannabis” and “neurological disorders,” “medical cannabis” and “amyotrophic lateral sclerosis,” “medical cannabis” and “Parkinson,” and “medical cannabis” and “Alzheimer”. The articles were thoroughly screened by reviewing each article with titles, abstracts, and content of the full articles. We only included the studies published between 1986 and 2021 and human studies (clinical trials, case reports, and published protocols) in the English language, including adults of 18 years of age and older, and we excluded review articles and position studies (Figure 1). An additional search on clinicaltrials.gov was also performed using “ALS” and “cannabis,” “Parkinson” and “cannabis,” and “Alzheimer” and “cannabis”.

Only sparse data on the benefits of medical cannabis in neurological disorders are available from clinical studies. As we know that cannabis is a complex plant with hundreds of phytocannabinoids, several components are studied in the following clinical studies. Tetrahydrocannabinol (THC) and cannabidiol (CBD) are the most studied in therapeutic use as they are the two major compounds of the *Cannabis sativa* L*.* plant. THC could be synthetized from CBD acid extracted from the plant as dronabinol. Nabilone is completely synthetized and is an analog of THC whereas Sativex^®^, which is a commercialized medication, contains a mix of THC and CBD directly extracted from the plant of cannabis (nabiximol).

To date, we have only found four clinical studies exploring the use of medical cannabis in ALS (Weber et al., 2010; Joerger et al., 2012; Meyer et al., 2019; Riva et al., 2019). Data from these studies are summarized in Table 1. The use of dronabinol alone did not demonstrate improvement in cramp intensity, cramp frequency, and fasciculation intensity neither on quality of life, sleep, appetite, and depression in a randomized controlled trial (RCT) of 2010 (Weber et al., 2010). The lack of treatment effect could be due to the short duration treatment (2 weeks). In parallel, an equilibrated mix of tetrahydrocannabinol (THC) and cannabidiol (CBD) in the oromucosal spray Sativex^®^ seems to be effective on ALS-related spasticity and on the patients’ global impression of change in a 6-week RCT (Riva et al., 2019) and also in a cohort study (Meyer et al., 2019). Noticeably, Sativex^®^ (an equilibrated mix of THC and CBD) is already commercialized and indicated for symptom improvement in adult patients with resistant spasticity due to multiple sclerosis. All these studies reported good tolerability of medical cannabis. Therefore, these modest but encouraging results suggest the need for further studies enrolling a higher number of patients.

**TABLE 1:** Summary of clinical studies exploring medical cannabis in ALS.

Reference	Study design	Number and the type of patients	Molecule explored and the route of administration	Dose/frequency duration	Outcomes and efficacy	Safety
Weber et al. (2010)	Randomized, double-blind, placebo-controlled crossover study	*n* = 22 ALS patients suffering from daily cramps (completed)	Dronabinol, Marinol^®^; per os	5 mg of dronabinol twice daily during 2 weeks Wash-out: 2 weeks	-No significant effect on cramp intensity (primary outcome)	Two AEs non–study-related: one pneumonia and one deep venous thrombosis
					-No significant effect on number of cramps per day, number of cramps during daytime and bedtime, fasciculations, quality of life, quality of sleep, appetite, and depression (secondary outcomes)	
Joerger et al. (2012)	Randomized, double-blind, placebo-controlled study	*n* = 9 ALS patients suffering from cramps	Dronabinol; per os	1) 5 mg (single dose)	-PK linear with a doubling of the AUC	-Drowsiness, euphoria, orthostasis, sleepiness, vertigo, and weakness: significantly more frequent in patients receiving 10 mg THC as compared to 5 mg THC per day
				2) wash-out: 2 weeks	-High inter-individual PK variability	-No association between drug exposure and the occurrence of AE
				3) 10 mg (single dose)	-Heart rate peaked approximately together with the plasma concentrations of THC-OH	
Meyer et al. (2019)	Observational, retrospective, monocentric, cross-sectional cohort study	*n* = 32 ALS patients suffering from spasticity	Mix of THC:CBD (2.7 mg:2.5 mg), Sativex^®^; oromucosal spray	three groups:	-Severe spasticity related to high doses of Sativex^®^	No AE reported
				>7 sprays (*n* = 11)	-High treatment satisfaction	
				<7 sprays (*n* = 16)	-*n* = 16 discontinued the treatment during observation period	
				Infrequent use (*n* = 5)		
Riva et al. (2019)	Randomized, double-blind, placebo-controlled study	*n* = 59 ALS patients	Mix of THC:CBD (2.7 mg:2.5 mg), Sativex^®^; oromucosal spray	14 days titration, duration 6 weeks	-Significant reduction of ALS-related spasticity	-three temporarily discontinuations of AEs
		Sativex^®^, *n* = 29			-Significant effect of the patient’s impression of change	one nausea and anxiety event, one influenza and accidental fall, and one disease progression event
		Placebo, *n* = 30			-No significant reduction of the global impression of change (caregivers and physicians), pain, spasm frequency, sleep, timed 10-m walk, scores on the amyotrophic lateral sclerosis functional rating scale—revised, forced vital capacity, scores on the barthel activities of daily living index, and body mass index	-No SAEs

AE, adverse event; ALS, amyotrophic lateral sclerosis; CBD, cannabidiol; SAE, severe adverse event; THC, tetrahydrocannabinol.

Concerning PD patients, eight clinical studies were published (Consroe et al., 1986; Frankel et al., 1990; Sieradzan et al., 2001; Carroll et al., 2004; Zuardi et al., 2009; Chagas et al., 2014a, 2014b; Lotan et al., 2014), as shown in Table 2. Medical cannabis could be effective both on motor symptoms (dystonia, dyskinesia, and fluctuations), and non-motor symptoms (anxiety, sleep quality, hallucinations, and disorientation) (Consroe et al., 1986; Frankel et al., 1990; Sieradzan et al., 2001; Zuardi et al., 2009; Chagas et al., 2014a; Lotan et al., 2014). Two studies (one RCT and one case report of five PD patients) show that there is not any reduction of motor and non-motor symptoms. One open-label study shows that there is an improvement of motor and non-motor symptoms only at the highest dose of CBD (400 mg/day for 4 weeks). Case reports of four PD patients show that there is an improvement of the quality of sleep without nightmares and reduction of agitation. Five studies show improvement of motor and non-motor symptoms and quality of life (three open-label studies and two RCTs). Anyway, all studies demonstrate that there are no serious adverse effects. The main limitations to these findings are short study duration and small sample sizes. Another limitation may be due to the low bioavailability of THC and CBD in oral preparations. This means that there is an obvious need for larger well-conducted studies.

**TABLE 2:** Summary of clinical studies exploring medical cannabis in PD.

Reference	Study design	Number and the type of patients	Molecule explored and the route of administration	Dose/frequency duration	Outcomes and efficacy	Safety
Consroe et al. (1986)	Open-label study	*n* = 5 PD patients with dystonia	CBD; per os	From 100 mg/day to 600 mg/day increased 100 mg/week, duration 6 weeks	Improvements in dystonia, dose-related in a 20–50% range	-four hypotension events
						-three exacerbations of hypokinesia and/or tremor with a higher dose of CBD
						-two dry mouth
						-two sedation events
						-two lightheadedness
Frankel et al. (1990)	Case report	*n* = 5 PD patients resistant to common therapies	Cannabis; smoking	1 g; 2,9% THC, duration not reported	No effects in reducing the tremor	Drowsiness and mild euphoria
Sieradzan et al. (2001)	Randomized, double-blind, placebo-controlled crossover study	*n* = 9 PD patients with L-Dopa–induced dyskinesia	Nabilone; per os	0,03 mg/kg, duration not reported	Significant improvement in L-Dopa dyskinesia	-Two AEs with withdrawn: one vertigo and one hypotension
						-Other AEs (*n* = 5 patients): mild sedation, “floating sensation”, dizziness, hyperacusis, partial disorientation, and visual hallucinations
Carroll et al. (2004)	Randomized, double-blind, placebo-controlled crossover study	*n* = 19 PD patients with L-Dopa–induced dyskinesia	Mix of THC:CBD (2.5 mg:1.25 mg); per os	Max 0.25 mg/kg/day THC, administration twice daily, during 4 weeks	-No significant effects reported on UPDRS, Rush, Bail, PDQ-39 scales	No SAE reported
				Wash-out: 2 weeks	-No significant effects in improving the quality of life	
Zuardi et al. (2009)	Open-label study	*n* = 6 PD patients with at least 3-month-old psychosis	CBD; per os	150 mg/day to 400 mg/day, duration 4 weeks	-Significant improvements in BPRS and psychotic symptoms, in sleep quality, less hallucinations, and disorientations (PPQ)	No AE reported
					-Significant improvement in UPDRS and CGI-I	
Chagas et al. (2014a)	Randomized, double-blind, placebo-controlled study	*n* = 21 PD patients without dementia or psychiatric symptoms and no occasional cannabis consumers	CBD; per os	75 mg/day or 300 mg/day, duration 6 weeks	Significant difference in PDQ39 between placebo and CBD 300 mg/day groups	No AE reported
Chagas et al. (2014b)	Case reports	*n* = 4 PD patients with sleep behavioral problems	CBD; per os	75 mg/day (*n* = 3); 300 mg/day (*n* = 1), duration 6 weeks	Four patients described an improvement in sleep behavioral disorders	No AE reported
Lotan et al. (2014)	Open-label study	*n* = 22 PD patients	Cannabis; smoking	0, 5 g, duration not reported	-Significant improvements in UPRDS, in tremor, in rigidity, and bradykinesia	-Two AEs: one hypoglycaemia and one dizziness
					-Significant improvement in sleep and pain scores	

BPRS, Brief Psychiatric Rating Scale; CBD, cannabidiol; CGI-I, clinical global impression–improvement; PD, Parkinson’s disease; PDQ-39, Parkinson’s disease questionnaire; THC, tetrahydrocannabinol; UPDRS, Unified Parkinson’s Disease Rating Scale.

To our knowledge, five RCTs and two open-label studies were published in AD regarding medical cannabis effectiveness and safety (Volicer et al., 1997; Walther et al., 2006; Mahlberg and Walther, 2007; Walther et al., 2011; Ahmed et al., 2015; Shelef et al., 2016; Herrmann et al., 2019). Results of these studies are available in Table 3. Only dronabinol and nabilone were experimented in AD patients. The benefits published in these studies were improving in agitation, nocturnal motor activity, disturbed behavior, anorexia, and the patient’s global impression of change (Volicer et al., 1997; Walther et al., 2006; Walther et al., 2011; Shelef et al., 2016; Herrmann et al., 2019).

**TABLE 3:** Summary of clinical studies exploring medical cannabis in AD.

Reference	Study design	Number and the type of patients	Molecule explored and the route of administration	Dose/frequency duration	Outcomes and efficacy	Safety
Volicer et al. (1997)	Randomized, double-blind, placebo-controlled crossover study	*n* = 15 AD patients with behavioral disorders and anorexia	Dronabinol; per os	2.5 mg twice daily, duration 6 weeks	-Significant improvement in body weight	-Nine tiredness and eight somnolence events
					-Significant improvement in the severity of behavioral disorders	Seven euphoria events
					-Significant improvement in the negative affect score	-No SAE reported
Walther et al. (2006)	Open-label study	*n* = 6 patients in the late stages of dementia and suffering from circadian and behavioral disorders	Dronabinol; per os	2.5 mg/day, duration 2 weeks	-Significant reduction in the nocturnal motor activity	No AE reported
					-Significant improvement of the NPI score (agitation, aberrant motor, and night-time behaviors)	
					-Significant reduction in appetite disturbances and irritability	
Mahlberg and Walther (2007)	Randomized, placebo-controlled study	*n* = 24 AD patients suffering from agitated behavior	Dronabinol; per os	2.5 mg/day, duration 2 weeks	-Significant reduction in nocturnal motor activity	No AE reported
					-Significant improvement of the NPI score	
Walther et al. (2011)	Randomized, double-blind, placebo-controlled crossover study	*n* = 2 AD patients with night time agitation	Dronabinol, Marinol^®^; per os	2.5 mg/day, duration 2 weeks	-Reduction in nocturnal motor activity	No AE reported
Ahmed et al. (2015)	Randomized, double-blind, placebo-controlled crossover study	*n* = 10 patients suffering from dementia	THC, Namisol^®^; per os	-Weeks 1–6: 0.75 mg twice daily	—	-Two AEs at 0.75 mg: one dizziness and one fatigue
				-Weeks 7–12: 1.5 mg twice daily		-Four AEs at 1.5 mg: three agitation and one fatigue
				Wash-out period: 4 days		-No SAEs
				Duration: 12 weeks		
Shelef et al. (2016)	Open-label study	*n* = 11 patients with dementia and NPS	THC; per os	-2.5 mg twice daily	-Significant improvement in CGI and NPI scores (delusions, agitation/aggression, irritability, apathy, sleep, and caregiver distress)	Three AEs: one fall, one confusion, and one dysphagia
				-5 mg twice daily if 2.5 mg ineffective		
				-7.5 mg twice daily if 5 mg ineffective		
				Duration: 4 weeks		
Herrmann et al. (2019)	Randomized, double-blind, placebo-controlled crossover study	*n* = 39 AD patients suffering from agitation	Nabilone; per os	1 mg/day; 1,5 mg/day; 2 mg/day according to tolerance, duration 14 weeks	-Significant improvement in agitation (CMAI)	-36 AEs: 22 sedation, eight falls, one bradycardia, one myoclonic jerk, one elevated urea level, one rash, one NPS increase, and one dizziness
					-Significant improvement in NPI (caregiver distress, behavior)	
				Wash-out: 1 week	-Significant improvement in the sMMSE score	-Five SAEs: two lethargy, one death, one high INR, and one myocardial infarction
					-Significant improvement in the nutritional status without weight gain	

AD, Alzheimer’s disease; AE, adverse event; CGI, clinical global impression of change; CMAI, Cohen-Mansfield agitation inventory; sMMSE, standardized mini-mental status examination; NPI, neuropsychiatric inventory; NPS, neuropsychiatric symptoms; SAE, severe adverse event, THC, tetrahydrocannabinol.

To resume, among these 19 clinical studies, nine were randomized double-blind placebo-controlled designed. Open-label design has inherent limitations of a placebo effect and rater bias. Moreover, as the experimental products and the routes of administration used were different (synthetic or natural and mix of cannabinoids or only one cannabinoid; per os or smoked), it adds an additional difficulty to compare results. According to the experimental product, it could also be difficult to perform a placebo-controlled design because of the conspicuous and characteristic smell of a cannabis cigarette, for example. It still underlines that more well-conducted studies would be necessary to further strengthen evidence of effectiveness. Nevertheless, these results are hopeful for patients suffering from these neurological disorders. Moreover, adverse effects reported with the use of medical cannabis do not seem to be limiting for its clinical use. Reported adverse effects were expected ones compared to the knowledge of cannabis use in general population (drowsiness, euphoria, sleepiness, weakness, dizziness, hypotension, and dry mouth). Due to pharmacokinetics variability of medical cannabis (and its numerous metabolites), future studies should apply parallel group study design rather than crossover design.

To date, 13 studies are registered in clinicaltrials.gov; two protocols are already published in Medline (Urbi et al., 2019a; Timler et al., 2020). Urbi et al. published a protocol of a randomized double-blind placebo-controlled study in ALS patients to evaluate the efficacy of a mix oil of CBD:THC (25 mg CBD: <2 mg THC) in slowing the disease progression. Secondary objectives are safety and tolerability. Timler et al. carried a randomized double-blind crossover study experimenting a mix oil of THC:CBD (3:2) in patients with dementia, on behavior symptoms, quality of life, and discomfort by pain.

Overall, clinical studies exploring medical cannabis in neurological disorders show different results depending on the methods and outcomes. Some show benefits on motor symptoms of neurological diseases, some on non-motor symptoms, and others no benefit at all. Therefore, it is becoming essential to conduct more and larger clinical studies in order to scientifically enlighten clinicians and first and foremost patients.

## What About Patients’ View?

As cannabis has been presented as a treatment for many medical conditions for few years, patients experiment this plant in many ways to manage their neurological disorders. Nevertheless, very few surveys have been conducted to describe 1) the consumers (medical condition and demographics); 2) the consumption (the cannabinoid type, form, route of administration, frequency, duration, and way of acquisition); 3) the relief symptoms (duration and level of the relief); 4) the adverse effects (type, duration, and frequency). It is unavoidable to understand the motivations and experiences of cannabis use among people living with neurological disorders to better orient clinical trials.

In 2004, Amtmann and colleagues published a worldwide anonymous web survey analyzing the answers of 131 ALS patients (Amtmann et al., 2004). The mean age was 54 years, and patients were mostly male (75%). Respondents reported a stable family life and a high education level for the majority. The median time since ALS diagnosis was 3 years, and the mean duration was 4 years. About 10% of the respondents (*n* = 13) reported the use of cannabis to relieve symptoms of ALS in the last 12 months. They mostly consume smoking cannabis. Only three of them reported using medical cannabis (dronabinol). Concerning relieve symptoms, patients reported cannabis as moderately effective in symptoms of appetite loss, depression, pain, spasticity, and drooling and ineffective in reducing difficulties with speech and swallowing and sexual dysfunction. The longest relief was reported for depression. In 2004, Venderova and colleagues also sent an anonymous questionnaire to all patients attending the Prague Movement Disorder Centre (Venderová et al., 2004). In total, 339 questionnaires were returned, and 25% of the respondents declared having taken cannabis. The mean age of cannabis users was 63.9 years, and the mean duration of PD was 8.3 years. They mostly reported an oral consumption once a day. Interestingly, none of them reported their doctor. PD patients described alleviations, especially in motor symptoms: 44.7% in bradykinesia, 37.7% in muscle rigidity, 30% in tremor, and 14.1% in L-dopa-induced dyskinesias. Another anonymous web survey managed on PD patients in the United States in 2020 analyzed the answers of 1,064 patients (Feeney et al., 2021). The mean age of the respondents was 71.2 years, male accounted for 52.5%, and the mean PD duration was 7.4 years. They were mostly highly educated with 78% of retired, evolving in a stable family life. About 25% of them reported the use of cannabis in the previous 6 months, and 35.6% of them considered themselves as regular users. They most frequently reported spraying or drooping, smoking, and eating as their primary method of cannabis use. The ways of acquisition were medical dispensary (38.7%) and family/friend gift (24.5%). When known, patients reported products with a high THC dosage in 21.2%, to get a better efficacy for both motor and non-motor symptoms. The reported relief symptoms were non-motor symptoms insufficiently controlled by classic medications: anxiety (45.5%), pain (44%), sleep disorders (44%), and specific motor symptoms such as stiffness (43%) or tremor (42%). Only 12.6% of PD cannabis users reported adverse effects (anxiety, impaired coordination, and dizziness). Interestingly, cannabis non-users (75.5%) reported two major reasons for not using cannabis: the lack of evidence (59.9%) and the fear of cannabis adverse effects (34.9%). In 2021, a German nationwide questionnaire survey described the used of medical cannabis in PD patients (Yenilmez et al., 2021). A total of 1,348 questionnaires were analyzed. The mean age of the patients was 71.6 years, and the mean PD duration was 11.6 years. Cannabis use was reported in 8.4% of the questionnaires, with a reduction of pain and muscle cramps in more than 40% of users (respectively, 43.9 and 41.4%). Moreover, more than 20% of them described an improvement in depression (28.1%), stiffness/akinesia (27.3%), sleep disorders (27.1%), freezing (25.0%), tremor (24.3%), anxiety (24.0%), and restless legs syndrome (21.4%). The improvements were related to 54.1% of oral CBD use and to 68.2% inhaling THC-containing cannabis. In the majority of patients (85%), cannabis was well-tolerated. Adverse effects reported were mainly fatigue, dizziness, and ravenous appetite. Another recent survey showed that 95% of movement disorders specialist neurologists reported to be asked to prescribe medical cannabis to their patients (Bega et al., 2017).

Concerning AD patients, a recent Polish anonymous web survey addressed to caregivers identified the attitudes and beliefs of caregivers of individuals with AD toward CBD oil in Poland (Leszko and Meenrajan, 2021). A total of 73 caregivers answered the questionnaire. They reported an effective use of CBD oil in behavioral symptoms of AD, to slow memory loss, agitation, anxiety, and insomnia. Most of the caregivers (84%) answered that CBD oil improved their care recipient’s quality of life. None of them reported adverse effects with the use of CBD oil. It is also interesting to note that only 63% of them informed their physician about this habit. In this survey, people also reported lack of information about the legacy, the medical use of cannabis as far as a lack of scientific data.

Despite being great sources of information, these surveys present several limitations. First, the results are based on small sample sizes compared to the affected population; respondents may not have been representative of the entire ALS, PD, and AD population. Second, internet users’ population constitutes a selection bias because all patients with neurological disorders could not use the internet, and internet users tend to be highly educated. Third, cannabis users may have been more inclined to answer the surveys and inflated the number of users and benefits, leading to a possible answer bias. Another limitation is the country of survey and/or residence because cannabis could be legal or not.

## What Are the Main Risks of Cannabis Use in Neurological Disorders?

The main risk is that most of the therapeutic uses of cannabis are performed outside of a medical pathway exposing patients to uncontrolled drugs (street cannabis) and unexpected drug–drug interactions. Published case reports show CBD interactions with antiepileptic drugs (Anderson et al., 2019; Gilmartin et al., 2021), warfarin (Grayson et al., 2018), immunosuppressants such as tacrolimus (Leino et al., 2019), and methadone (Madden et al., 2020). In all these case reports, the consequence of the drug–drug interaction was an increase in plasma concentrations of co-administered medications and potential associated complications. Cannabis could also cause well-known psychiatric adverse effects (psychosis, paranoia, anxiety, disorientation, etc.). Therefore, it is important to supervise and regulate the consumption of medical cannabis. In the majority of clinical studies exploring medical cannabis, exclusion criteria included history of psychiatric disorders (Collin et al., 2007; Wallace et al., 2015; van de Donk et al., 2019). In addition to the respiratory adverse effects, cardiovascular complications are poorly known but also reported with the use of cannabis. Jouanjus et al. conducted an observational retrospective study in 2011 in patients admitted to a French hospital with a relation of cannabis use. In total, 200 patients were included, and 619 adverse effects were reported with 9.5% of cardiovascular ones (Jouanjus et al., 2011). Serious cardiovascular complications described with the use of cannabis are arrythmia including ventricular tachycardia, acute coronary syndromes, peripheral complications (arteriopathies), and cerebral complications (acute cerebral angiopathy, transient cortical blindness, and spasm of the cerebral artery) (Jouanjus et al., 2014; Goyal et al., 2017). There is a lack of consensus that likely reflects a general knowledge gap and paucity of data to guide clinical practice. Nevertheless, it is essential to supervise cannabis consumption and consider the medical history and the concomitant medication use in these patients to avoid serious complications.

## Discussion/Conclusion

In recent decades, the endocannabinoid system has attracted considerable interest as a potential therapeutic target in numerous pathological conditions. Medical cannabis has clearly demonstrated several benefits on neurological disorders, owing to its pleiotropic pharmacological activity. Preclinical, clinical, and real-life experiences described in ALS, PD, and AD are even more important cues to develop research on medical cannabis. Acceptable safety and tolerability profiles are also strong arguments to be considered in the development of medical cannabis. It is now essential to answer several questions to broadly develop medical cannabis in neurological disorders: 1) which cannabinoids (THC, CBD, mix of THC and CBD, and others)? 2) What dosage? 3) What frequency? 4) Which route of administration? 5) In which symptoms? Answers to these questions will be helpful for patients and clinicians to manage care pathways with medical cannabis treatment.

As all pharmacological substance and a fortiori with its pleiotropic activity, clinicians should be cautious with the use of medical cannabis because of drug–drug interactions and with the medical history of their patients. Only few patients inform their clinicians of cannabis experience, and very few data are available on these interactions.

Nevertheless, there is a call for more clinical studies to secure cannabis consumption in a medical enrollment. As far as patients and patients’ associations are calling for it, there is an urgent need to manage clinical studies to provide stronger evidence and secure medical cannabis use. Therefore, more controlled clinical studies with larger neuropsychiatric populations should be prioritized to bring important cues in the near future and support the translation of research findings to clinical settings.
